# The Influence of Land Use on the Grassland Fire Occurrence in the Northeastern Inner Mongolia Autonomous Region, China

**DOI:** 10.3390/s17030437

**Published:** 2017-02-23

**Authors:** Yiping Li, Jianjun Zhao, Xiaoyi Guo, Zhengxiang Zhang, Gang Tan, Jihong Yang

**Affiliations:** 1 Provincial Laboratory of Resources and Environmental Research for Northeast China, Northeast Normal University, Changchun 130024, China; li0p578@nenu.edu.cn (Y.L.); zhaojj662@nenu.edu.cn (J.Z.); guoxy914@nenu.edu.cn (X.G.); 2 Jilin Surveying and Planning Institute of Land Resources, Changchun 130061, China; tangang156@163.com (G.T.); yangjh123@hotmail.com (J.Y.)

**Keywords:** land use, grassland fire, human activity, land use degree

## Abstract

Grassland, as one of the most important ecosystems on Earth, experiences fires that affect the local ecology, economy and society. Notably, grassland fires occur frequently each year in northeastern China. Fire occurrence is a complex problem with multiple causes, such as natural factors, human activities and land use. This paper investigates the disruptive effects of grassland fire in the northeastern Inner Mongolia Autonomous Region of China. In this study, we relied on thermal anomaly detection from the Moderate Resolution Imaging Spectroradiometer (MODIS) sensor to identify fire occurrences, and land use data were acquired by Landsat Thematic Mapper/Enhanced Thematic Mapper (TM/ETM). We discussed the relationship between land use and the spatial distribution of grassland fires. The results showed that the impact of land use on grassland fires was significant. Spatially, approximately 80% of grassland fires were clustered within 10 km of cultivated land, and grassland fires generally occurred in areas of intense human activity. The correlation between the spatial distribution of grassland fires and the land use degree in 2000, 2005 and 2010 was high, with R^2^ values of 0.686, 0.716, 0.633, respectively (*p* < 0.01). These results highlight the importance of the relationship between land use and grassland fire occurrence in the northeastern Inner Mongolia Autonomous Region. This study provides significance for local fire management and prevention.

## 1. Introduction

Grassland is one of the Earth’s ecosystems and one of the most widely distributed vegetation types on the planet. China, with nearly 400 million hectares of grassland, which accounts for 40 percent of the total land area in China and 3.7 times its cultivated land area, is one of the countries with the most abundant grassland resources in the world [1]. Fire is one of the most important interference factors in natural ecosystems [2]. Grassland fires can cause losses to grassland ecosystems and human life, such as affecting the economy in pastoral areas and endangering the safety of people in pastoral areas. In northeastern China, grassland covers a vast area, and grassland fire is a common phenomenon. Nearly one sixth of this area experiences serious fire damage every year. In 1950–2000, more than 50,000 fires occurred in Northeast China [3], and they affected an area of 2 million hectares and caused a loss of 1.5 million dollars in pastoral areas [4]. Moreover, the occurrence of grassland fire is a very complex problem that is affected by many driving factors, among which human activities and land use play very important roles in its occurrence.

Until now, many studies around the world have demonstrated the strong spatial relationship between wildfires and land use [5,6,7,8,9,10,11,12,13]. In the tropics, several studies regarding the possibility of vegetation fire occurrence have shown that land use change caused by some inducing conditions (climate, topography and applicability of specific vegetation types) and human activities is the decisive factor in vegetation fire occurrence, and the accessibility to roads is an important determinant of forest fires [5,6,7]. In the Amazon region, precipitation and temperature affect the fire occurrence and frequency directly, and from a spatial perspective, the fire occurrence is directly related to road networks and land use, for the fires were used to clear forests and maintain extensive pastures and farmland [8,9]. With land use changes (increases in the number of roads and the infrastructure) caused by human activities, fires occurred at an increasingly frequent rate. The human activities contained forest crushing, logging and some previous burning, which easily increased the fire risk [9]. In the eastern Amazon region, fires occurred more frequently closer to the edges of forests [8,9,10]. In Spain, anthropogenic activities have been shown to cause fires [11]. The exploitation of land by humans leads to changes in forest land use that can increase the abundance of shrubs and other flammable vegetation types. As land use types change, fires can become more frequent and widespread [12,13,14,15]. In some areas of the United States and northern Mexico, climatic conditions and land use changes caused by human activities both alter the fire frequency. The effects of climate on fire occurrence vary in different periods, and land use has a very important and constant effect on the fire occurrence [16,17]. Fires occurred frequently within 1 km of cultivated land and roads (70%) [18]. In the European Mediterranean basin, temperature and precipitation have become less important, and most fires are currently related to human activities. In areas where the intensity of human activities is high, the intensity of land use is large, and grassland fires are more common [19,20,21]. Human-induced fires have a long history, and human activities have considerably changed fire scenarios [22,23,24]. On the global scale, some studies have analyzed the relationship between fire occurrences, the vegetation structure and land use and noted that natural fire sources are uncommon on the global scale. However, the impacts of human activities and land use on fire occurrence considerably vary globally, and most fires are caused by human activities; thus, fire frequency is closely related to the land use intensity [25]. Fire plays multiple roles in land use, not only as a hunting tool but also it is used to clear forests, maintain grasslands, control pests and manage crops. These human activities increase the fire risk, which is easy to cause fire occurrence in the forest fringe, and then lead to fire disaster [18,24,25].

In Inner Mongolia of China, arid and semi-arid grassland fire is also a frequent occurrence annually due to human activities and more than 90% fires occurred in spring and autumn [26]. Many studies have shown that the fire occurrence is closely related to human activities. When we discuss the root cause of fire occurrence, we cannot ignore the influence of human activities on fire [26]. Several studies have also shown that the closer the distance between villages and roads, i.e., areas in which human activities are more intensive, the more frequently fires occur. Especially in rural areas, vehicles, smoking, burning stubble and grass on fields, worship, children playing with fire, cooking and other human activities, are more likely to cause fire [2,27]. Some studies have also considered the effect of human activities on the spatial distribution of fires, and the results demonstrated that the spatial distribution of fires is closely related to human activities [28,29,30].

All of the above studies indicated that in addition to climate, human activity is a large influence on the fire occurrence. For the spatial distribution and the intensity of human activities can be expressed by land use and land cover, the proximity of the land use is one of the main causes of the wildland fire occurrence.

Human activities, as the most important factors that affect the fire occurrence, are difficult to measure directly. The land use degree reflects the development and utilization of land resources by humans. Therefore, the degree of land exploitation reflects the socioeconomic status of a region [31,32,33,34]. Early research methods of the land use degree mainly included two types of systems. One type is based on the type of system, such as the generalization of the land use intensity proposed by the German scientist Von Thunen, as well as the general land use pattern, diagrammatic model, land use polarization and antipolarization pattern and gravity pattern methods proposed by other scholars [35]. These models were mainly formed by analysing the social and natural factors that affect land use. The other type is expressed by quantitative index systems, such as China’s indirect index system. In the book “Land Use Planning” [36], several land use indicators are used to reflect the degree of land use. These early research methods also reflected the influence of humans on the land use degree to a certain extent. Land use is a vast classification system, and the quantification of the land use degree is based on the upper and lower limits. The upper land use limit is the peak utilization of land resources by humans. At this limit, further exploitation is not possible. The lower limit of land use is the starting point of the human exploitation and utilization of land resources [37]. Thus, the quantification of the land use degree effectively represents the extent of land exploitation associated with human activities. The socioeconomic factors that affect land use change include population change, technology development, economic growth, the affluence degree, value orientation, the demand for land products, land investment, the urbanization degree, land use policies and so on [32,33,38,39]. Thus, land use change in an area is greatly influenced by human activities. As a resource, we concerned more about the degree of land use and the change in this degree. In the case of regional land use, the comprehensive index of land use is generally used to reflect the extent of land use in a given period. The significance of the comprehensive index of land use is that it can reflect the intensity of regional land use. The degree of human development and utilization of land also characterizes the effects of land use caused by human factors [32,33,40]. The above studies all show that the land use degree reflects the extent of land development, and the driving factors of land use and land cover change are mainly associated with human activities.

The objective of this paper is to evaluate the effects of human activities on grassland fire occurrence by analyzing the relationship between land use and grassland fires. The land use degree was introduced to characterize the comprehensive development level of regional land use and to analyse the impact on grassland fire in the northeastern Inner Mongolia Autonomous Region, Northeast China.

## 2. Study Area, Data and Methods

### 2.1. Study Area

**Figure 1:**
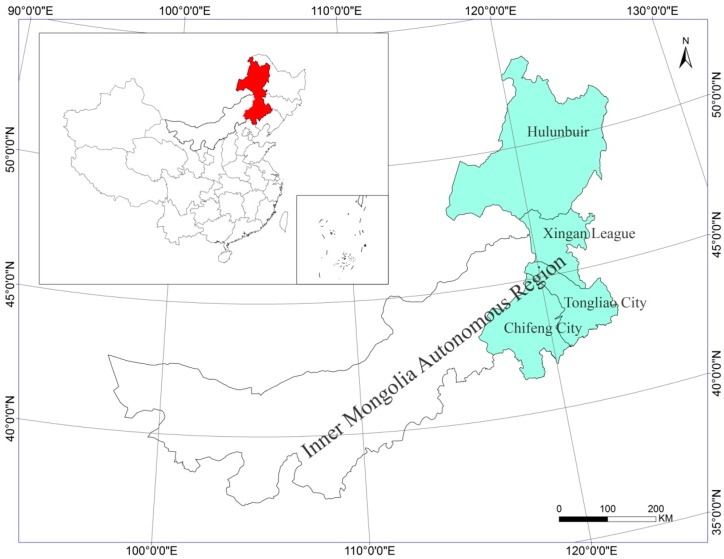
The location of the study area in the Inner Mongolia Autonomous Region, Northeast China.

The study area is located in the northeastern Inner Mongolia Autonomous Region in Northeast China, which is situated between 41.2–53.23 N and 115.22–126.06 E (Figure 1). It is approximately 1348 km long and 703 km wide and covers a total area of 452,540 km^2^. There are four municipalities in the study area, including Hulunbuir, Hinggan League, Chifeng City, and Tongliao City, which contain 36 counties. The population was 12.27 million in 2005. The northeastern portion of the study area is adjacent to Mongolia and Russia. 

On average, 451 grassland fires occur in this region every year and cover an area of approximately 8203 km^2^. These fires cause considerable economic losses and are mainly caused by human factors. The study area has a typical temperate continental monsoon climate, including a cold temperate zone and middle temperate zone. Additionally, extreme changes in temperature occur between summer and winter. The rainfall in the area is irregular, and the majority of annual precipitation occurs in summer. The grassland coverage in this area is up to 42.34%. From 2000 to 2010, the average annual decrease in grassland area was approximately 123.82 km^2^, and most of the grassland has been converted into urban residential and industrial lands, with a smaller percentage converted to arable land.

### 2.2. Data

#### 2.2.1. Grassland Active Fire Data

Currently, most of the world’s fire occurrence is obtained through acquiring remote sensing data for effective assessment and management [41,42,43]. Most of the fire data comes from MODIS active fire data, MODIS active fire data is especially designed for fire management. At present, it is available free and commonly used. Many scholars have used the MODIS active fire product data to assess the relationship between fire and land use accurately [18,44,45]. MODIS has a fast response system, and its fire detecting algorithm shows excellent performance with a high accuracy for fire records [46,47].

In this study, grassland active fire data were provided by MODIS products with a 1 km spatial resolution. The MODIS data include daily thermal anomalies calculated in the 4 μm and 10 μm bands. The fire hotspot series, which is part of the collection five temporal thermal analysis active fire dataset, includes data from both the Terra and Aqua satellites. MOD14A1 (provided by Terra) and MYD14A1 (provided by Aqua) are daily level 3 fire hotspot products based on a sine curve projection, and the classifications are shown in Table 1.

We processed the MODIS images using the MODIS Re-projection Tool (MRT), which included mosaicking, re-projection, and Albers Equal area projection. Table 2 shows the MRT parameters and output type. MODIS active fire data covered a series of 13-year fire events (2000–2012) and this data was extracted from the stacked result of layer with the level 7, 8, 9 and converted into vector points. These points were clipped by grassland layers and saved as shapefile layers.

#### 2.2.2. Land Use Data

In this study, the land use data were provided by the Data Center for Resources and Environmental Sciences, Chinese Academy of Sciences (RESDC) (http://www.resdc.cn) and included land use in 2000, land use in 2005 and land use in 2010. The land use data were classified into seven categories: cultivated land, grassland, forest land, residential land, urban land, water (rivers and lakes) and unused land. The land use data was obtained from the Landsat TM/ETM images (30 m spatial resolution) by supervised classification [48,49,50].The data were produced every five years.

### 2.3. Methods

#### 2.3.1. Land Use and Grassland Fires

Because a year of land use cannot represent long-term land use, we used land use in 2000, 2005, and 2010 to represent the following three periods of land use data: 2000–2002, 2003–2007, and 2008–2012, respectively. We divided the grassland active fire points into three periods: 2000–2002, 2003–2007, and 2008–2012. Cultivated land, water, rural residential land and urban land were extracted from three periods of land use data to obtain the number of fire points at different buffer distances for the land use types in 1 km increments. The relationship between land use and the spatial distribution of grassland fires was analysed using a regression analysis.

#### 2.3.2. Land Use Degree and Grassland Fires

To further explore the impact of land use on grassland fires, this paper introduces the land use degree, which mainly reflects the breadth and depth of land use. Additionally, this metric reflects the degree of human utilization and exploitation of land to a certain extent. In this paper, a quantitative comprehensive weight index was used to characterize the land use degree in different regions. Liu et al. [31] proposed a method for the analysis and evaluation of land use degree. They divided the land use types into four levels and assigned different level index. The associated formula is as follows [31]:

$$La=100\times \overset{n}{\underset{i=1}{\sum }}\left(A_{i}\times C_{i}\right)\;La\in \left(\;100,\;400\right).$$

where *La* is index of the comprehensive land use degree, *A_i_* is the level *i* index of land use, *C_i_* is the percentage of the level *i* land use in a moving window (9 × 9), and *n* is the number of land use classifications. In my study, based on the above standards of land use index and the geographical and human condition of the study area, we obtained the assignment table of the classification index of land use in the study area. Then we used the quantitative evaluation of the comprehensive land use degree index to carry out the operation and research. Table 3 shows the classification index of the land use.

We calculated the land use degree in 2000, 2005, and 2010; the land use degree per unit area; and the number of fire points per unit area in the 36 administrative districts. The relationship between the land use degree and grassland fire occurrence was determined using a regression coefficient.

#### 2.3.3. Land Use Changes and Grassland Fires

Spatial errors exist in point data, and they cannot reflect the conditions of continuous space. Therefore, we converted the point data into spatially continuous surface data. In this paper, the spatial distribution of grassland fires was estimated based on a kernel density function. Kernel density estimation is a method of reconstructing a probability density function from some sampling points as follows [51]:

$$\lambda \tau \left(s\right)=\overset{n}{\underset{i=1}{\sum }}\frac{1}{\tau^{2}}k\left[\frac{\left(s-s_{i}\right)}{\tau }\right].$$

where λτ(s). is the density of the spatial distribution of the corresponding variable at s points and $s_{1}$, ...., $s_{n}$ are the geographical distribution densities of corresponding variables. *τ* is bandwidth, which was used to define the degree of smoothness, and *s* is the centre of a circle with a given radius. The greater *τ* is, the higher the degree of smoothness. The function *k* is the kernel function of a bivariate probability density. We acquired the spatial distributions of the densities of different scale variables by adjusting the value of the bandwidth, which was eventually set to 25 km.

The change in the land use degree from 2000 to 2005 was obtained by subtracting the 2005 land use degree from the 2000 land use degree, and this process was repeated between 2005 and 2010. The change in the grassland fire density was acquired in the same way. The land use degree and fire density raster data were reclassified separately. The pixels were assigned values of (−1, 0, 1) if the land use degree and fire density decreased, remained constant, or increased, respectively. Then, we extracted the spatial pixels in which the land use degree changed and analysed the relationship between the changes and the corresponding grassland fire density. If the grassland fire density was positively related to the land use degree, it represented the same trend; otherwise, it represented the opposite trend. 

## 3. Results

### 3.1. Land Use and Grassland Fires

#### 3.1.1. The Spatial Distribution of Grassland Fires

**Figure 2:**
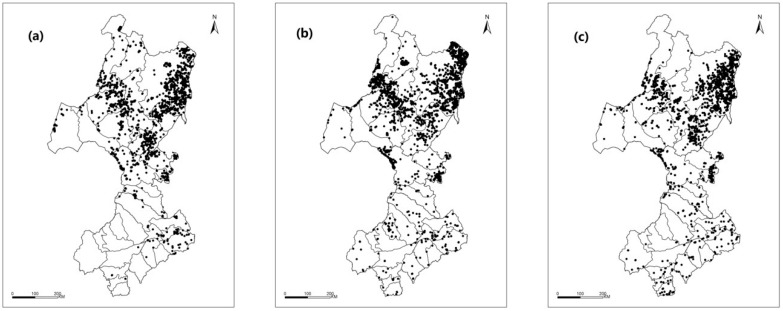
Active grassland fire events in the study area: (**a**) 2000–2002; (**b**) 2003–2007; (**c**) 2008–2012.

The spatial distribution of active grassland fires within the three periods is shown in Figure 2. A total of 1766, 2306 and 1791 active grassland fires occurred from 2000 to 2002, from 2003 to 2007, and from 2008 to 2012, respectively. The number of fire events fluctuated in different periods, but the spatial distribution remained nearly unchanged. Most active fires were distributed in the north of the study area, and fewer were observed in the south of the study area.

A statistical analysis showed that the number of grassland fires varied minimally in each administrative region. Among them, the administrative area with the highest number of active grassland fires was the Oroqen Autonomous Qi, which experienced 432 fires from 2000–2002, 706 fires from 2003 to 2007, and 579 fires from 2008 to 2012. 

Some counties had more than 100 fires in the three periods, including Yakeshi Shi, MolidawaZizhiqi, HorqinYouyiQianqi, Ergun Shi and others. In the southern part of the study area, the number of grassland fires did not reach 10 in the three study periods in most counties, such as Harqin Qi, Hure Qi, Linxi County, BairinYouqi, ArHorqin Qi, Ulanhot Shi, Tuquan County and others. The number of active grassland fires in each administrative region is shown in Table 4.

#### 3.1.2. The Monthly and Seasonal Distribution of Grassland Fires

**Figure 3:**
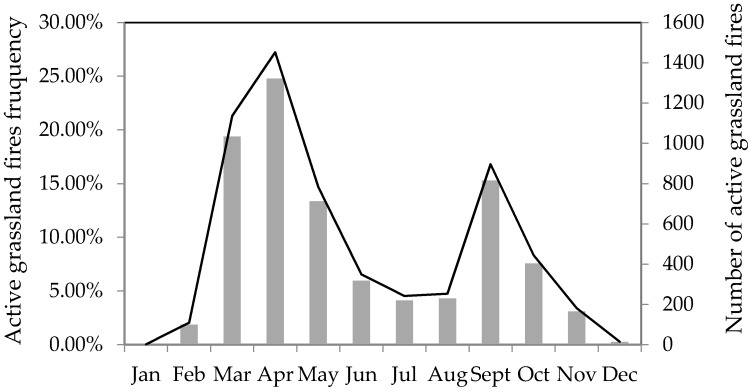
The annual distribution of active grassland fires in the study area. The line shows the number of active grassland fires; the bar shows the active grassland fires frequency.

Grassland fires in the study area varied greatly in each year. As shown in Figure 3, the highest frequency of grassland fires occurred in April, with the frequency of 24.77%, followed by March and September, with frequencies of 19.38% and 15.28% respectively. The frequencies of May and October were also relatively high, which were 13.35% and 7.57% respectively. While the grassland fires frequencies in February, June, July, August and November were relatively low, and In January and December, there were almost no grassland fires.

**Figure 4:**
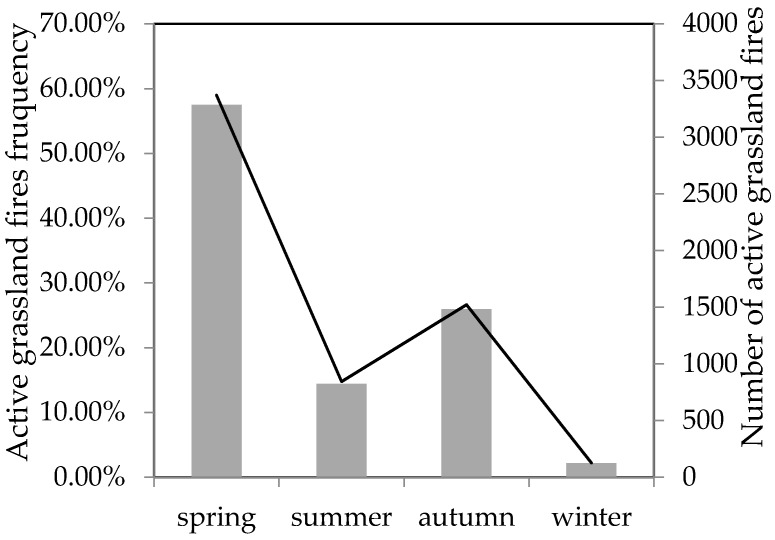
The seasonal distribution of active grassland fires in the study area. The line shows the number of active grassland fires; the bar shows the active grassland fires frequency.

When we studied the seasonal time rule of grassland fire in the study area, we divided the season according to climatological classification method, that is, March to May is spring, June to August is summer, September to November is autumn, December to February is winter, and the results are shown in Figure 4. The grassland fire frequency was the highest in spring, reaching 57.5% of the whole year, and the grassland fire frequency was the lowest in winter, accounting for 2.15% of the annual fire occurrence. The study area is cold in winter, and the grassland is covered with snow for a long time and there are few human activities out of doors. The water content of grassland is high in summer, which is not good for the occurrence and spread of grassland fire. Therefore, the grassland fire frequency was relatively low in summer and winter. While the spring and autumn are suitable periods for human activities out of doors, human activities occur frequently, such as the farming in the spring and the sacrifice in the autumn, which have created the conditions for the grassland fires occurrence.

#### 3.1.3. Land Use Analysis

**Figure 5:**
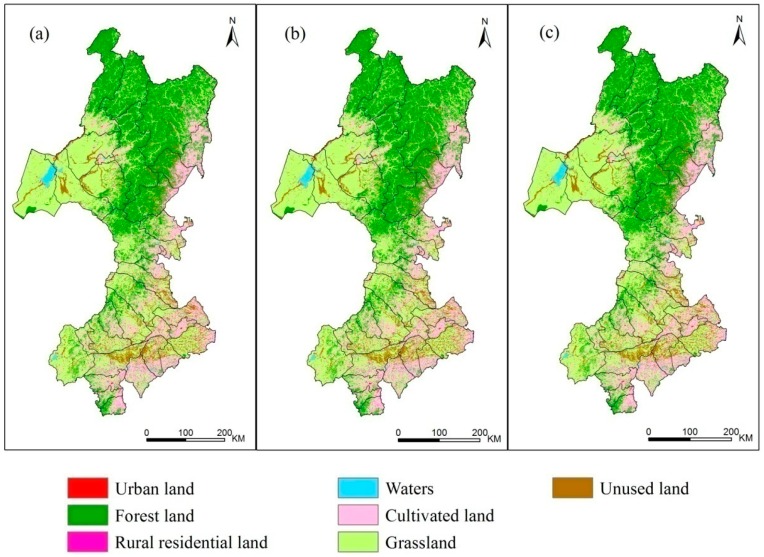
Land use classification results in the study area: (**a**) land use in 2000; (**b**) land use in 2005; (**c**) land use in 2010.

The spatial distribution of the land use data (2000, 2005, and 2010) in the study area is shown in Figure 5.

In the study area, grassland comprised the largest area, with more than 190,000 km^2^. This area decreased a little from 2000 to 2010, but the proportion of grassland area remained at more than 40% of the total study area in this period. The proportions of forest area in 2000, 2005 and 2010 were 33.46%, 33.61% and 33.59%, respectively. The cultivated land area in 2000, 2005 and 2010 comprised 69,304 km^2^, 69,780 km^2^, 70,006 km^2^, respectively, and the corresponding percentages were 15.31%, 15.42% and 15.47%. The proportions of water and unused land comprised more than 15% of the study area. The proportions of rural residential land and urban land in 2000, 2005 and 2010 did not exceed 1% of the total area, and the area of human construction land was small.

Statistics show that the area of each land use type varies from 2000 to 2010, but the change in each land use type is less than 1%. Although the areas of cultivated land, urban land and unused land all increased, the increase in the cultivated land area was the largest. The area of cultivated land increased by 467 km^2^ and 226 km^2^ in the 2000–2005 and 2005–2010 periods, respectively. The total increases in urban land and unused land were 71 km^2^ and 153 km^2^, respectively, from 2000 to 2010. The area of grassland has decreased since 2000, and it decreased by 939 km^2^ and 423 km^2^ in the 2000–2005 and 2005–2010 periods, respectively. The areas of rural residential land and forest land increased by 17 km^2^ and 706 km^2^, respectively, from 2000 to 2005, while the areas of rural residential land and forest land decreased by 6 km^2^ and 100 km^2^, respectively, from 2005 to 2010. However, the area of water decreased by 309 km^2^ from 2000 to 2005 and increased by 128 km^2^ from 2005 to 2010. The specific changes in land use area are shown in Table 5.

#### 3.1.4. Land Use and the Distribution of Grassland Fires

The relationship between the number of grassland fire points and the distance from four land use types in 2000, 2005 and 2010 were analysed separately. The results are shown in Table 6. The distributions of urban land, rural residential land and water and grassland fire points showed good linear fitting relationships. The R^2^ values between rural residential land and the spatial distribution of grassland fires were 0.708, 0.737 and 0.725 (2000, 2005 and 2010). The R^2^ values between water and the spatial distribution of grassland fires were 0.898, 0.889 and 0.824 (2000, 2005 and 2010). The R^2^ values between urban land and the spatial distribution of grassland fires were relatively low at 0.588, 0.598 and 0.656 (2000, 2005 and 2010). The relationship between cultivated land and the spatial distribution of active grassland fires was well represented by inverse model fitting, with R^2^ values of more than 0.9, reflecting a relatively high correlation.

**Figure 6:**
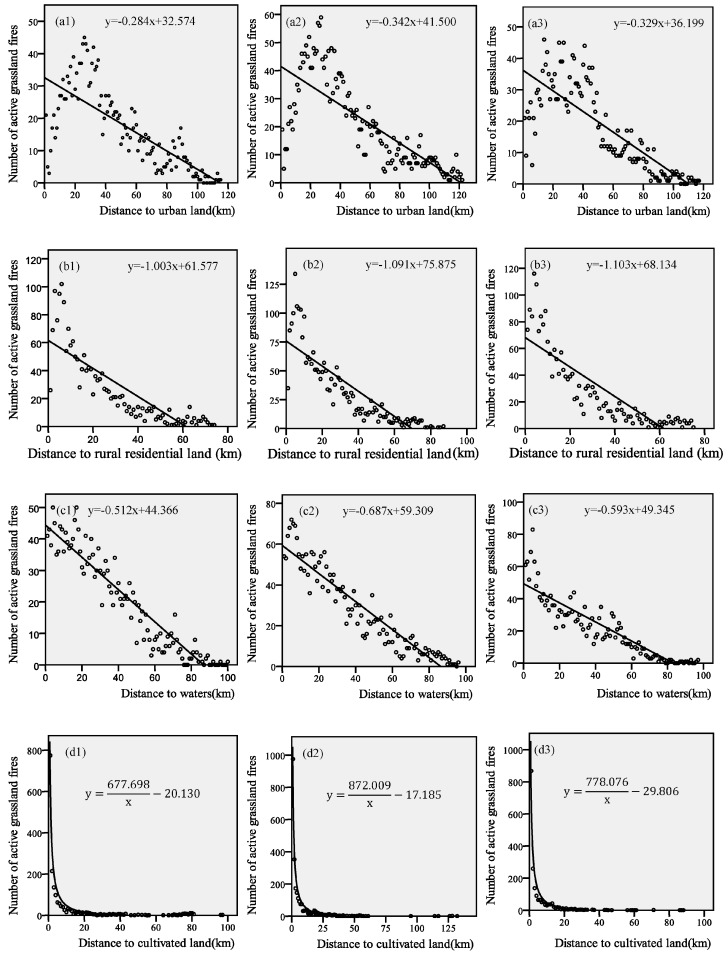
Effects of various land uses on the spatial distribution of grassland fires: (**a1**–**a3**) urban land (2000–2010); (**b1**–**b3**) rural residential land (2000–2010); (**c1**–**c3**) cultivated land (2000–2010); (**d1**–**d3**) water (2000–2010).

The trends associated with urban land, rural residential land, cultivated land and water were relatively consistent with the spatial distribution of grassland fires in 2000, 2005 and 2010. Additionally, there was a strong correlation between the spatial distribution of grassland fires and these four land uses. The closer the distance to the four land uses, the more frequently grassland fires occurred. Figure 6 shows that land use exhibited a certain degree of change over time, and the impact of land use on the spatial distribution of grassland fires remained strong. The R^2^ value of the relationship between urban land and the spatial distribution of grassland fires was relatively low. The number of grassland fires within 15 km of urban land was relatively low, and beyond 15 km, the number of grassland fires decreased with increasing distance from urban land. In 2000, approximately 79% of all grassland fires occurred within 10 km of cultivated land. As the area of cultivated land year increased annually, the number of grassland fires within 10 km of the cultivated land also increased. In 2005 and 2010, more than 80% of fires occurred within this distance. Although the relationship between the distance from cultivated land and the spatial distribution of grassland fires was nonlinear, it was quite striking. The relationships between the spatial distribution of grassland fires and rural residential land and the spatial distribution of grassland fires and water can be described well by linear fitting at three points in time. The fitting coefficient between the four land uses and the spatial distribution of grassland fires varied moderately in different years. The relationships between the spatial distribution of grassland fires and the four land uses in all three periods were significant.

### 3.2. Land Use Degree and Grassland Fires

#### 3.2.1. The Spatial Distribution of the Land Use Degree

**Figure 7:**
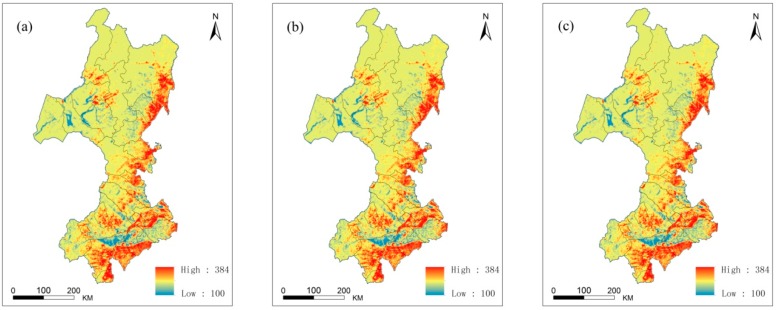
Land use degree in the study area: (**a**) land use degree in 2000; (**b**) land use degree in 2005; (**c**) land use degree in 2010.

Figure 7 shows the spatial distribution of the land use degree in 2000, 2005 and 2010. In general, the land use degree in the southern part of the study area was generally higher than that in the northern part. From 2000 to 2010, the areas of urban land and residential land with high land use degrees increased slowly. The increase in the total area was only 82 km^2^, and its annual growth was less than 10 km^2^. Because human construction land accounted for only approximately 1% of the total area of the study region, the change in the land use degree was small. Statistics showed that the land use degrees in different administrative regions exhibited small changes. The land use degrees per unit area of 36 administrative regions in 2000, 2005 and 2010 are shown in Table 7. The land use degrees per unit area in different administrative regions exhibited considerable differences. XinBaragZouqi exhibited one the smallest land use degrees per unit area of all administrative regions. The largest land use degree per unit area was observed in the Ulanhot Shi, with a land use degree per unit area of more than 270 in the three periods. The land use degrees per unit area of Tongliao Shi and MolidawaZizhiqi were second only to that of the Ulanhot Shi. These degrees per unit area all exceeded 250, and the land use degrees per unit area in the other most administrative units were all between 200 and 240.

#### 3.2.2. The Relationship between the Land Use Degree and Active Grassland Fires

We discuss the relationship between the land use degree per unit area and grassland fires per unit area in 36 administrative regions based on a regression analysis to reveal the effects of anthropogenic activities on grassland fires. The results are shown in Table 8. 

The impacts of the land use degree on grassland fires were statistically significant at the significance level of 0.01. The three R^2^ values between the land use degree (2000, 2005 and 2010) per unit area and grassland fires (2000–2002, 2003–2007 and 2008–2012) per unit area were 0.683, 0.716 and 0.633 (2000, 2005 and 2010), respectively. These studies proved that the greater the land use degree, the more frequently grassland fires occurred. Additionally, the greater the degree of land exploitation due to human activities, the more frequently grassland fires occurred, which suggests that human activities influenced grassland fires indirectly. This paper introduced the land use degree to analyse the factors that affect grassland fires, and this metric has a certain guiding significance for future grassland management.

### 3.3. Land Use Changes and Grassland Fires

**Figure 8:**
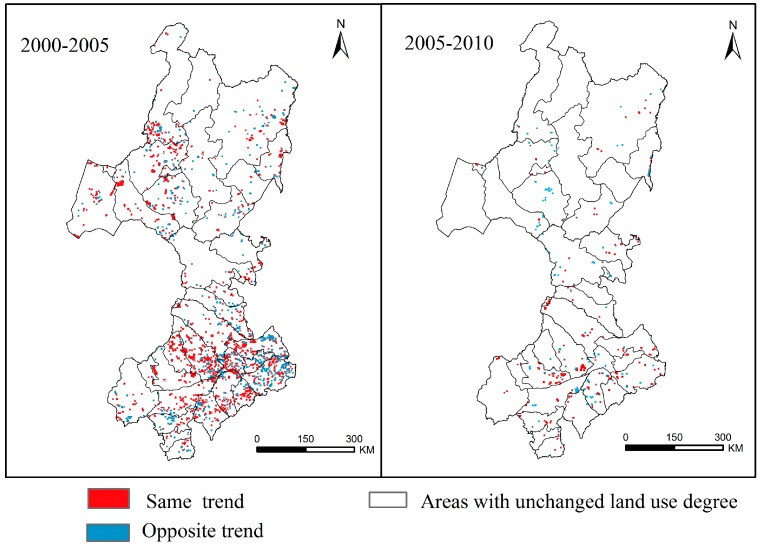
Change in the land use degree in different years: from 2000 to 2005 (**left**) and from 2005 to 2010 (**right**).

In this study, the change in the regional comprehensive land use degree was used to characterize the impact of land use change on grassland fires. Over time, the land use degree in the study area changed to a certain extent. From 2000 to 2005, the increase in the land use degree per unit area in the study area was 7.68, and the increase in the land use degree per unit area from 2005 to 2010 was 5.48. The change in the land use degree per unit area from 2000 to 2005 was relatively large and mainly distributed in the southern and northwestern parts of the study area, such as the Hulunbuir grassland. From 2005 to 2010, the area of land use change per unit area was relatively small and mainly concentrated in the southern part of the study area. The land use change in the northern area was also small, and the specific changes are shown in Figure 8. The increased land use degree was characterized by the continuous development and utilization of land by humans. We analysed the changes in fire density in areas where the land use degree changed. An obvious correlation between the fire density and the land use degree can be observed in most areas.

From a spatial perspective, we identified the pixels in which the fire density changed in areas with land use degree changes. The results showed that from 2000 to 2005, the number of spatial pixels in which both the land use degree and fire density exhibited the same trend accounted for 63.72% of all pixels. From 2005 to 2010, this percentage was 60.60%. Thus, as the land use degree of an area increased (decreased), the fire density also increased (decreased). It is obvious that the change in the land use degree had a considerable influence on the fire occurrence. Additionally, changes in the land use degree were mainly associated with human factors and land disturbances. Thus, these relationships characterize the effect of human activities on the occurrence of grassland fires to a certain extent.

## 4. Discussion

The results of this paper suggest that there is a strong spatial association between regional land use and the spatial distribution of grassland fires. The closer the distance to urban land, rural residential land, cultivated land or water, the more frequently grassland fires occurred. These land use types are associated with areas where human activities and disturbances are more intense. In the study area, farming and grazing played dominant roles in the industrial structure. Notably, human activities are particularly intense in cultivated land areas, and approximately 80% of all active grassland fires occurred within 10 km of cultivated land. In the areas surrounding the cultivated land, several human activities, including smoking, harvesting, burning stubble and others, can easily cause fires that can spread to grasslands [4]. In this study, it was also proved that there was a strong relationship between the grassland fires occurrence and the spatial distribution of water. Water is also a region of intensive human activities. Around this land use type, the human activities are more likely to lead to fire occurrence [52,53]. Within 15 km of urban land, the grassland distribution is relatively limited, and land exploitation is relatively severe. Thus, grassland fires occur less in these areas. Fires are caused by human activities and land use. Therefore, this study provides theoretical support for grassland management. However, in the three years, land use patterns in the study area corresponded to the number of grassland fires. Although the annual changes in land use were small, some differences might affect the analysis results. Additionally, the effects of land use on the spatial distribution of grassland fires were analysed only by considering distance and without considering topography or other factors that might affect the analysis results to various extents.

Meanwhile, the land use degree in the study area was calculated based on land use data. The classification of land use data influenced the land use degree directly. This study obtained a significant positive correlation between the comprehensive land use degree and the occurrence of grassland fires. The land use data in the study area were obtained from Landsat TM/ETM (30 m spatial resolution). Therefore, these data were relatively accurate [54,55]. In addition, we did not consider the relationships between the sub-classes under the current land use categories and the grassland fire distribution. Moreover, the classification of land use in this region is not detailed. Therefore, the scales of urban land, rural residential land and other land use types were not discussed. The population and the level of socioeconomic development varied in different urban land and rural residential areas, and these variations may affect the research results.

In this study, only statistical analyses of pixels were considered to analyse the impact of the change in the land use degree on grassland fire occurrence. From 2000 to 2005, the land use degree per unit area increased by 7.68, but it only increased by 5.48 from 2005 to 2010. The percentage of pixels with similar trends between the fire density and land use degree in different years exceeded 60%. The total grassland density in areas with an increased land use degree from 2000 to 2005 was 72.852. In addition, the increase in the land use degree from 2005 to 2010 was small, and the sum of the corresponding fire density values in pixels increased by only 0.043 These values suggest that the grassland fire density changes were consistent with the changes in the intensity of the land use degree. In this region, the change in the land use degree reflects a change in the intensity of human activities. Meanwhile, the results showed that the effects of human disturbances on grassland fires varied with the intensity of human activities.

In this study, active grassland fire points were based on MODIS hotspot data with a 1 km spatial resolution. The minimum detectable fire size was 10 ha; thus, fires of less than 10 ha were not recorded. Grassland fires occur for a short time and the surface temperature decreases fast. Additionally, active grassland fire points were obtained at four specific times during the day and night, so small fire events at other times were excluded from the analysis because of the time sampling method or detection issues, such as the presence of cloud cover [56,57,58]. In addition, the grassland covers a relatively vast area in this study region, which contains a variety of grassland types. We generalized the fire points in different grassland types into one category, so we did not specifically analyse the relationship between land use and fire points in different types of grassland. The degree of human disturbance varied with different types of grassland, so the extent of grassland utilization was unequal, which impacted the scale of grassland fires. These problems have a certain influence on the analysis results. 

The fire occurrence is affected by many factors, and many studies have shown that climatic and fuel factors have an important effect on the fire occurrence [59,60,61,62,63,64,65]. However, we only analysed the effect of land use on grassland fires. In this study area, the latitude difference between the north and the south is relatively large, and the study area is a typical temperate continental monsoon climate, including a cold temperate zone and middle temperate zone. Empirically, climate is a common factor that influences the fire occurrence; however, this paper only analysed the impact of land use associated with human disturbance on grassland fires, and climate differences were not discussed. These factors affect the results of the relationship between land use and grassland fires and which lead to the low correlation.

## 5. Conclusions

In order to evaluate the effects of human activities on grassland fire occurrence, the relationship between land use and grassland fires was analyzed in this article. The land use degree was introduced to characterize the comprehensive development level of regional land use and to analyse the impact caused of the comprehensive development of regional land use on grassland fires in the northeastern Inner Mongolia Autonomous Region, Northeast China. This study showed that the spatial distribution of grassland fires were clustered around cultivated land, rural residential land, urban land and water, where the human activities are intense. The relationship between land use degree and fire occurrence is positive and with the high land use degree in a region, the probability of grassland fire occurrence is large.

Generally, land use is a common influential factor in such studies. We will discuss the scales of urban land, rural land, roads, and other land use types in the future. Additionally, we will introduce social and economic factors to analyse the occurrence of fire in further detail. A more detailed classification of land use should provide a more accurate assessment of grassland fires because we depend only on a broad classification of land use in this study. All of these factors emphasize the importance of continuous studies. Introducing Geographically Weighted Regression or more sophisticated spatial autocorrelation approaches may further explain the spatial distribution of grassland fires. These are all essential needs in future studies.

## Figures and Tables

**Table 1:** The classifications of MOD14A1/MYD14A1 data set.

Grades	Representative Content
0	Untreated pixel
2	Untreated pixel
3	Water
4	Clouds
5	No fire bare
6	Unknown pixel
7	Low confidence level fire point
8	Medium confidence level fire point
9	High confidence level fire point

**Table 2:** The MRT parameter and output type table.

Parameter	Output Type
Projection type	Albers Equal Area
Projection coordinate system	WGS-1984
Spatial re-sampling resolution	1000 m
Data output format	Geo TIFF
Parameter save format	*.prm

**Table 3:** Classifications index of land use.

Classifications of Land Use Degree	Land Use/Land Cover	Classification Index
Classifications of unused land	Unused land	1
Classifications of grassland, forest land and water	forest land, grassland, water (rivers, lakes)	2
Classifications of Cultivated land	Cultivated land	3
Classifications of artificial surface	Urban land, rural residential land	4

**Table 4:** Number of active grassland fires in per administrative region.

NAME	Number of Grassland Fires		
	2000–2002	2003–2007	2008–2012
Ningcheng County	0	4	16
Harqin Qi	6	9	6
Chifeng Shi	2	4	14
Aohan Qi	0	7	11
Hure Qi	0	2	3
Ongniud Qi	1	5	4
Naiman Qi	6	8	18
Hexigten Qi	0	10	10
HorqinZuoyiHouqi	34	24	34
Linxi County	0	1	1
Tongliao Shi	16	14	12
Kailu County	8	16	5
BairinYouqi	0	3	2
BairinZuoqi	0	23	0
HorqinZuoyiZhongqi	11	10	17
ArHorqin Qi	0	8	2
Jarud Qi	11	11	17
Hulingol Shi	1	2	5
HorqinYouyiHouqi	18	9	18
Tuquan County	1	3	6
Ulanhot Shi	0	2	2
Jalaid Qi	105	25	55
HorqinYouyiQianqi	128	194	126
Zalantun Shi	140	60	101
EwenkizuZizhiqi	51	54	25
Hailar	5	3	8
Manzhouli Shi	0	9	11
XinBaragYouqi	29	11	19
Arun Qi	95	67	58
XinBaragZuoqi	24	54	24
Chen Barag Qi	116	122	86
MolidawaZizhiqi	184	255	235
Yakeshi Shi	128	198	132
Oroqen Autonomous Qi	432	706	579
Genhe Shi	38	81	5
Ergun Shi	176	292	124

**Table 5:** Land use change between 2000 and 2010 in the northeastern Inner Mongolia Autonomous Region.

Land Use	2000		2005		2010		2000–2005	2005–2010
	Area (km^2^)	%	Area (km^2^)	%	Area (km^2^)	%	Area (km^2^)	Area (km^2^)
Rural residential land	4100	0.91%	4117	0.91%	4111	0.91%	17	−6
Urban land	779	0.17%	814	0.18%	850	0.19%	35	36
Forest land	151,413	33.46%	152,119	33.61%	152,019	33.59%	706	−100
Cultivated land	69,304	15.31%	69,780	15.42%	70,006	15.47%	476	226
Grassland	191,689	42.36%	190,750	42.15%	190,327	42.06%	−939	−423
Water	6647	1.47%	6338	1.40%	6466	1.43%	−309	128
Unused land	28,608	6.32%	28,622	6.32%	28,761	6.36%	14	139

**Table 6:** The relationships between each land use type and the distribution of grassland fires by regression analysis (land use in 2000, land use in 2005, land use in 2010).

Year	Variables	Urban Land	Rural Residential Land	Cultivated Land	Water
2000	R^2^	0.588 **	0.708 **	0.928 **	0.898 **
2005	R^2^	0.598 **	0.737 **	0.961 **	0.889 **
2010	R^2^	0.659 **	0.725 **	0.937 **	0.824 **

** Correlation is significant at the 0.01 level (2-tailed).

**Table 7:** Land use degree per unit area in administrative region.

NAME	Land Use Degree Per Unit Area		
	2000	2005	2010
Ningcheng County	240.57	240.09	240.21
Harqin Qi	233.17	233.11	233.17
Chifeng Shi	245.31	244.89	245.12
Aohan Qi	247.52	247.30	247.25
Hure Qi	228.56	227.02	226.84
Ongniud Qi	204.05	204.66	204.50
Naiman Qi	211.80	213.74	213.30
Hexigten Qi	200.20	199.99	199.98
HorqinZuoyiHouqi	202.76	203.20	202.85
Linxi County	223.74	223.69	223.72
Tongliao Shi	256.28	256.23	256.38
Kailu County	238.91	239.82	239.97
BairinYouqi	202.38	202.63	203.12
BairinZuoqi	228.64	230.07	230.04
HorqinZuoyiZhongqi	225.92	227.87	228.01
ArHorqin Qi	207.30	208.57	209.07
Jarud Qi	210.57	210.80	211.06
Hulingol Shi	210.92	213.64	216.35
HorqinYouyiHouqi	207.40	206.95	206.97
Tuquan County	241.69	241.15	241.15
Ulanhot Shi	273.66	273.71	273.91
Jalaid Qi	225.04	225.15	225.32
HorqinYouyiQianqi	213.16	213.28	213.37
Zalantun Shi	205.41	205.50	205.56
EwenkizuZizhiqi	193.08	193.57	193.66
Hailar	231.68	231.24	231.46
Manzhouli Shi	197.64	198.02	198.70
XinBaragYouqi	193.53	193.27	193.27
Arun Qi	220.78	220.75	220.75
XinBaragZuoqi	190.77	190.11	190.13
Chen Barag Qi	197.63	198.18	198.19
MolidawaZizhiqi	251.86	251.42	250.80
Yakeshi Shi	203.55	203.56	203.56
Oroqen Autonomous Qi	203.56	203.41	203.44
Genhe Shi	199.18	199.22	199.22
Ergun Shi	201.77	201.88	201.89

**Table 8:** Correlations between the land use degree and the distribution of grassland fire.

Variables	Degree of Land Use 2000	Degree of Land Use 2005	Degree of Land Use 2010
R^2^	0.686 **	0.716 **	0.633 **

** Correlation is significant at the 0.01 level (2-tailed).
